# Ptomaines and Leucomaines

**Published:** 1888-12

**Authors:** 


PTOMAINES AND LEUCOMAINES; OR, THE PUTREFACTIVE AND PHYSIOLOGICAL ALKALOIDS. By Victor C. Vaughn, Ph. D., M. D., * * * and Frederick S. G. Novy, M. S., * * * of the University of Michigan. Philadelphia: Lea Bros. & Co. 1888.

The above is the modest title of a little book of the very highest scientific and professional value. It is an attempt to collect, arrange and systematize the facts recently discovered concerning basic substances formed during the putrefaction of organic matter, and others which are produced by the normal tissue changes in the living organism.

It has been known from the earliest times, that the eating of putrid flesh was liable to affect the health more or less seriously, and in many instances it produces fatal poisoning. Of the nature of these poisons comparatively little was known. It was supposed that putrefaction was simply oxidation and chemical changes of the dead substance when freed from the influence of vitality.

Microscopic observation demonstrated the fact that countless myriads of minute organisms were constantly present, engaged, it was supposed, in transforming the matter from the organic to other inorganic forms. Chemistry has succeeded in isolating a large number of products resulting from putrefaction, possessing the character of alkaloids and determining their physiological and toxicological properties. To these have been given the name ptomaines (from the Greek word, *ptoma*, a dead body). Of these there are probably several hundred; not, however, all of them poisonous. More than a score of them are exceedingly so.

Many of these are occasionally found in the food in ordinary use, such as oysters, sausage, ham, canned meats and fruits of all kinds, cheese, milk, ice-cream and bread.

It is impossible, in the brief space allowed us, to give a synopsis of the many points of importance established or suggested in this work. It is, however, a matter of so much importance that we earnestly recommend its purchase and careful perusal to our readers.

